# “First-person view” of pathogen transmission and hand hygiene – use of a new head-mounted video capture and coding tool

**DOI:** 10.1186/s13756-017-0267-z

**Published:** 2017-10-30

**Authors:** Lauren Clack, Manuela Scotoni, Aline Wolfensberger, Hugo Sax

**Affiliations:** Division of Infectious Diseases and Hospital Epidemiology, University Hospital Zurich, University of Zurich, Raemistrasse 100, CH-8091 Zurich, Switzerland

**Keywords:** Video, Transmission risk, Hand hygiene, Observation

## Abstract

**Background:**

Healthcare workers’ hands are the foremost means of pathogen transmission in healthcare, but detailed hand trajectories have been insufficiently researched so far. We developed and applied a new method to systematically document hand-to-surface exposures (HSE) to delineate true hand transmission pathways in real-life healthcare settings.

**Methods:**

A head-mounted camera and commercial coding software were used to capture ten active care episodes by eight nurses and two physicians and code HSE type and duration using a hierarchical coding scheme. We identified HSE sequences of particular relevance to infectious risks for patients based on the WHO ‘Five Moments for Hand Hygiene’. The study took place in a trauma intensive care unit in a 900-bed university hospital in Switzerland.

**Results:**

Overall, the ten videos totaled 296.5 min and featured eight nurses and two physicians. A total of 4222 HSE were identified (1 HSE every 4.2 s), which concerned bare (79%) and gloved (21%) hands. The HSE inside the patient zone (*n* = 1775; 42%) included mobile objects (33%), immobile surfaces (5%), and patient intact skin (4%), while HSE outside the patient zone (*n* = 1953; 46%) included HCW’s own body (10%), mobile objects (28%), and immobile surfaces (8%). A further 494 (12%) events involved patient critical sites. Sequential analysis revealed 291 HSE transitions from outside to inside patient zone, i.e. “colonization events”, and 217 from any surface to critical sites, i.e. “infection events”. Hand hygiene occurred 97 times, 14 (5% adherence) times at colonization events and three (1% adherence) times at infection events. On average, hand rubbing lasted 13 ± 9 s.

**Conclusions:**

The abundance of HSE underscores the central role of hands in the spread of potential pathogens while hand hygiene occurred rarely at potential colonization and infection events. Our approach produced a valid video and coding instrument for in-depth analysis of hand trajectories during active patient care that may help to design more efficient prevention schemes.

## Background

Healthcare-associated infections, including surgical site infections, ventilator-associated pneumonia, urinary tract infections, and catheter-associated bloodstream infections, prolong length of hospital stay and increase cost, morbidity and mortality [1–3]. Additionally, antibiotic resistance is emerging worldwide as a serious health threat [4].

Transmission of potential pathogens between patients occurs primarily via healthcare worker (HCW) hands when hand hygiene (HH) is omitted at critical moments [5, 6]. Such hand-to-surface exposures (HSE) occur frequently [7], resulting each time in a bi-directional exchange of microorganisms between the hand and the touched surface [6]. In consequence, hands transport microorganisms sequentially between surfaces [6]. Depending on the nature of the microorganisms and of the receiving surface, this can result in patient harm. If microorganisms feature antibiotic resistance, their transmission to a patient can result in prolonged carriage. If the microorganisms are virulent and the receiver surface is a skin lesion or an invasive device such as a central venous line, the transmission may result in healthcare-associated infection.

Several studies show that infectious microorganisms can survive on human skin long enough to be cross-transmitted and that hand hygiene using alcohol-based handrub is an effective way to decrease this transmission [8, 9]. With the WHO “My five moments for hand hygiene”, a user-centered concept based on education, training, monitoring and reporting of hand hygiene has been introduced with the goal to bridge the gap between scientific evidence and daily healthcare practice [10]. Yet, HCWs still fail to consistently apply hand hygiene. The lack of awareness regarding what people touch during their routine work may play an important role in this failure to adhere to established rules [11]. Today’s gold standard to monitor HH performance consists of direct observation of healthcare workers by trained observers during patient care [5, 12–14]. This method may not capture every HSE during fast-paced care and thus, underestimates the true risk of pathogen transmission [7, 15]. On the other hand, automated electronic hand hygiene monitoring systems still fall short of detecting all hand hygiene opportunities [16].

To better understand the nature of microbial hand-transmission in a real-life intensive healthcare setting, we built and pilot-tested a new observation and coding system that would consistently capture every HSE, and thus allow to study true transmission risks via HCWs’ hands.

## Methods

### Setting up and offsite-testing of the system

We opted for a mobile, head-mounted action camera (GoPro® Hero 4 Black edition, GoPro Inc., San Mateo, CA) worn by HCW during patient care. The camera was positioned on the forehead of the HCW by means of a head-strap and was oriented facing slightly downwards. With the help of an iPad mini (Apple, Cupertino, CA) the researcher could control the optimal orientation of the camera through a Wi-Fi connection. The camera was oriented to keep the participant’s hands in its visual field. In a first round, we tested and adjusted the camera in the medical high-fidelity simulator of our institution. After resolving all technical issues, we proceeded to videotape real-life care activity in three intensive care units (ICUs) specialized in trauma, cardiology, and visceral surgery at the University Hospital Zurich (USZ), Switzerland. The USZ is a 900-bed university-affiliated tertiary care center with a well-established infection prevention and control (IPC) group, weekly IPC rounds, and a designated IPC nurse consultant for each hospital ward.

### Participants and onsite-use of the system

A convenience sample of 10 participants was recruited among ICU nurses and physicians. Each participant wore the head-mounted camera during his/her morning shift for about 70 min. Morning shifts were chosen purposefully to guarantee that patient care activity took place. Subsequently, HCW continued their care activity without further interruptions by the researcher, who left the area.

### Video coding

**Fig. 1:**
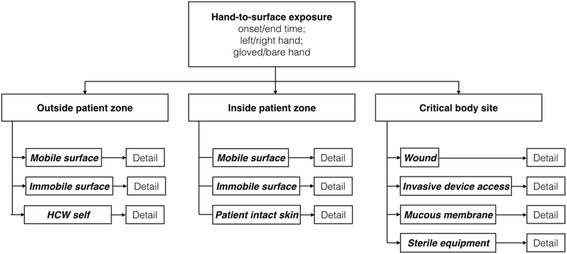
Hierarchical coding system. Legend: HCW self, healthcare workers touching themselves; one hand touching the other hand of the same HCW was not considered

The videos were exported from the camera and stored on a secured server. Episodes of ~30 min direct patient care were purposefully selected from each of the 10 videos for further processing. Within each of these video episodes, the occurrence, duration, and type of every HSE was systematically coded by a trained coder (MS) and supervised by a second person (LC) using the behavioral observation software INTERACT® (Mangold international, Arnstorf, Germany) together with a structured, hierarchical coding system (Fig. 1).

**Fig. 2:**
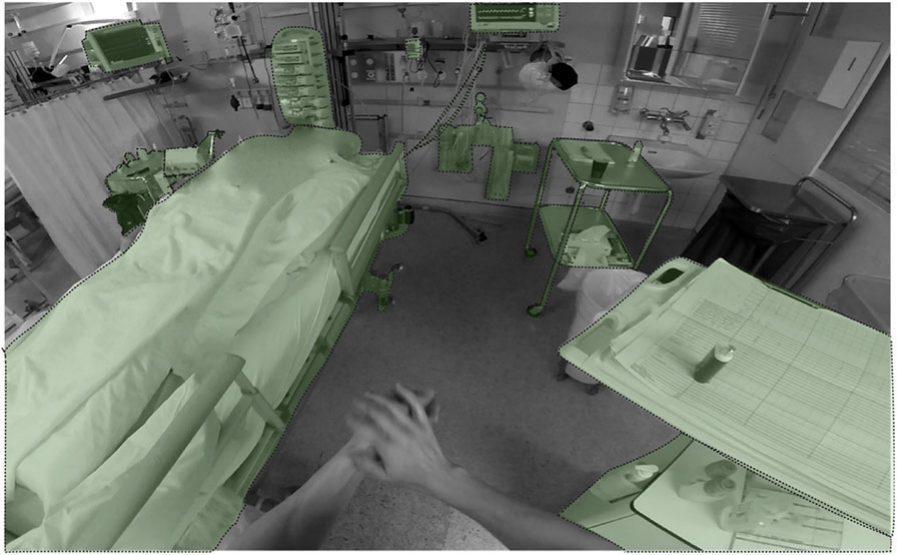
Typical visual field of the head mounted GoPro® action camera and color-coded patient zone. Legend: This screenshot demonstrates the first-person view recorded from the head camera. Objects and surfaces belonging to the patient zone are colored with a green overlay and dotted outline

The observation and coding system aimed to capture the duration and nature of all HSE, defined as contact between the observed healthcare worker’s hand and any other surface. The hierarchical coding system consisted of 4 levels, of which the first two indicate the nature of the hand (gloved vs. bare and right vs. left), and the latter two indicate the nature of the surface (location relative to patient zone and type of surface) involved in the hand-to-surface exposure (Fig. 1). In line with the WHO patient zone concept [10] and observation method [15] the third coding level distinguished between surfaces “inside patient zone”, “outside patient zone”, and “critical sites”. “Inside patient zone” was defined as the patient him−/herself and all items in the immediate environment likely to be colonized with patient flora [10]. The “outside patient zone” contained other patients with their respective zones, the HCW’s own body and professional apparel (“HCW Self”), and all the other areas and surfaces outside the patient zone [10]. “Critical sites” included clean sites such as medical devices or patient’s body parts that have to be protected against microbial colonization in order to avoid infections [10]. Hand hygiene actions were registered as specific events and coded as either “hand washing” or “hand disinfection” with alcohol-based handrub. Patient zones were established a-priori for each ICU setting to ensure accurate and consistent coding (Fig. 2).

### Data analysis

To assess the utility of the observation and coding system, we performed a descriptive analysis of frequency and duration of HSE. Coded event data were exported as comma separate values (.cvs) files, merged and edited in Excel (Microsoft, Redmond, WA) and analyzed in STATA special edition 12.0 (StataCorp, College Station, TX). Sequential analysis was additionally conducted to identify HSE sequences of particular relevance to infectious risks, as informed by the WHO ‘Five Moments for Hand Hygiene’ [10]. We defined sequences of touching a surface outside the patient zone followed by touching any surface inside a patient zone as a ‘*colonization event*’ and a sequence of touching any surface, except a critical site, followed by touching a critical site as an ‘*infection event’* (Table 1)**.** A *colonization event* would correspond to a modified WHO “Five Moments” concept’s Moment 1 “Before touching a patient” but include touching any surface inside the patient zone and not only the patient. This modification of Moment 1 was made to capture more precisely colonization risk of the patient by hospital flora that is brought into the immediate vicinity of the patient and from there to the patient. An *infection event* would correspond to WHO “Five Moments” concept’s Moment 2 “Before clean/aseptic procedure”. According to Sax et al., “Critical sites for infectious risks” included breaks in the patient’s intact skin such as wounds and catheter insertion sites, any patient mucous membrane, invasive devices in-situ if the lumen was accessed such as vascular or urinary catheters, and semi-critical or critical medical devices ready to be used on the patient [10].

**Table 1:** Events associated with the risk of patient cross-colonization or infection

	Origin HSE surface		Destination HSE surface
Patient Colonization Event; corresponding to WHO moment 1^a^	Any HSE outside patient zone	➔	Any surface inside patient zone (including fomites and intact patient skin, excluding critical sites)
Examples	Door handle, keyboard of mobile computer	➔	Patient bedside monitor, patient arm
Patient Infection Event; corresponding to WHO moment 2^a^	Any HSE (except the same critical site as arrival HSE surface)	➔	Any critical site
Examples	Patient arm, bedside monitor	➔	Central venous catheter insertion site, wound, sterile needle to be used on the patient

Legend: *HSE* hand-to-surface exposure. The symbol ➔ denotes the direct sequence of two HSE. ^a^WHO moment 1 with the modification that touching a surface inside the patient zone with or without touching the patient counts as Patient Colonization Event

## Results

**Fig. 3:**
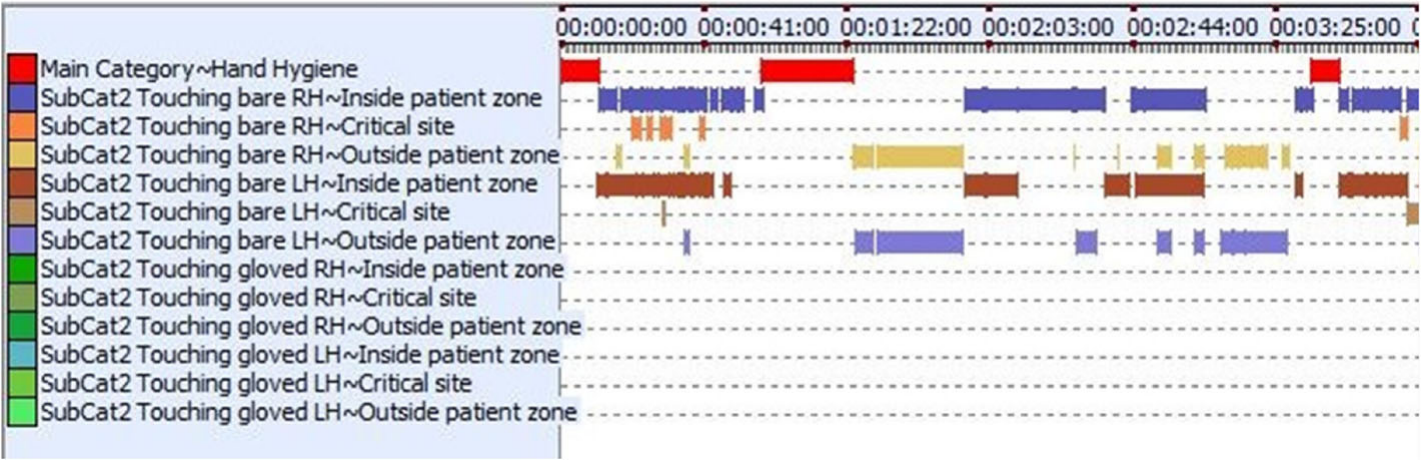
Timeline chart of video #7. Legend: An excerpt of the coding timeline from video #7. X-axis: time from 0:00–3:25 minutes. Y-Axis from top to bottom: Hand hygiene action, hand-to-surface exposure (HSE) patient zone bare right hand inside, HSE to critical site with bare right hand, HSE outside patient zone with bare right hand, HSE inside patient zone with bare left hand, HSE at critical site bare left hand, HSE outside patient zone with bare left hand, HSE inside patient zone with gloved right hand, HSE to critical site with gloved right hand, HSE outside patient zone with gloved right hand, HSE inside patient zone with gloved left hand, HSE at critical site with gloved left hand, HSE outside patient zone with gloved left hand

The 10 active care video sequences totaled 296.5 min and featured eight nurses of whom seven were female and two physicians of whom one was female, all right handed. Overall, 4222 HSE occurred, translating in an overall density of 14.2 HSE per minute or one HSE every 4.2 s. Exemplarily, Fig. 3 demonstrates the coding timeline of all HSE and hand hygiene actions in the first 3 min of video #7. Details on the frequency and nature of HSE and hand hygiene actions overall and per each video sequence appear in Table 2.

**Table 2:** Hand-to-surface exposures and hand hygiene actions

Video	#1	#2	#3	#4	#5	#6	#7	#8	#9	#10	Overall
ICU specialty	Trauma	Trauma	Trauma	Trauma	Cardio-surgery	Cardio-surgery	Cardio-surgery	General surgery	Cardio-surgery	General surgery	
Length of coded care sequence; min:sec	34:50	34:50	34:50	36:20	31:17	32:38	33:31	16:39	32:19	16:32	296:30
Gender	M	F	F	F	F	F	M	M	F	F	
Profession	N	N	N	N	N	N	N	P	N	P	
HSE; n	494	472	314	495	474	671	553	176	526	47	4222
HSE density; n/min	14,2	13,6	9,0	13,6	15,2	20,6	16,5	10,6	16,3	2,8	14,2
Mean HSE duration (SD); sec	8.9 (16.0)	6.8 (10.0)	11.7 (17.0)	8.1 (18.5)	7.0 (14.9)	5.3 (10.5)	5.6 (11.8)	11.7 (15.1)	6.8 (10.7)	11.8 (26.5)	7.44 (14.1)
Hand											
Right hand (%)	273 (55.3)	254 (53.8)	173 (55.1)	277 (56.0)	250 (52.7)	358 (53.4)	304 (55.0)	93 (52.8)	292 (55.5)	25 (53.2)	2299 (54.5)
Left hand (%)	221 (44.7)	218 (46.2)	141 (44.9)	218 (44.0)	224 (47.3)	313 (46.7)	249 (45.0)	83 (47.2)	234 (44.5)	22 (46.8)	1923 (45.6)
Gloves worn during HSE											
No (%)	355 (71.9)	420 (89.0)	221 (70.4)	214 (43.2)	406 (85.7)	671 (100)	334 (60.4)	176 (100)	474 (90.1)	47 (100)	3318 (78.6)
Yes (%)	139 (28.1)	52 (11.0)	93 (29.6)	281 (56.78)	68 (14.4)	0	291 (39.6)	0	52 (9.9)	0	904 (21.4)
Any surface inside patient zone (% of all HSE)	289 (58.5)	222 (47.0)	196 (62.4)	133 (26.9)	131 (27.6)	134 (20.0)	225 (40.7)	122 (69.3)	278 (52.85)	45 (95.7)	1775 (42.0)
Patient intact skin (% of HSE inside patient zone)	12 (4.2)	30 (13.5)	12 (6.1)	13 (9.8)	0	15 (11.2)	45 (20.0)	8 (6.6)	29 (10.4)	14 (31.1)	178 (10.0)
Mobile object inside patient zone (% of HSE inside patient zone)	241 (83.4)	163 (73.4)	130 (66.3)	112 (84.2)	117 (89.3)	80 (59.7)	168 (74.7)	109 (89.3)	234 (84.2)	29 (64.4)	1383 (77.9)
Immobile surface inside patient zone (% of HSE inside patient zone)	36 (12.5)	29 (13.1)	54 (27.6)	8 (60.2)	14 (10.7)	39,829.1)	12 (5.3)	5 (4.1)	15 (5.4)	2 (4.4)	214 (12.1)
Any surface outside patient zone (% of all HSE)	148 (30.0)	220 (46.6)	89 (28.3)	350 (70.7)	322 (67.9)	506 (75.4)	115 (20.8)	53 (30.1)	148 (28.1)	2 (4.3)	1953 (46.3)
HCW own body (outside patient zone) (% of HSE outside patient zone)	7 (4.7)	36 (16.4)	25 (28.1)	47 (13.4)	60 (18.6)	107 (21.2)	79 (68.7)	50 (94.3)	28 (18.9)	0	439 (22.5)
Mobile object outside patient zone (% of HSE outside patient zone)	114 (77.0)	160 (72.7)	49 (55.1)	235 (67.1)	175 (54.4)	346 (68.4)	18 (15.7)	3 (5.7)	92 (62.2)	2 (100)	1194 (61.1)
Immobile surface outside patient zone (% of HSE outside patient zone)	27 (18.2)	24 (10.9)	15 (16.9)	68 (19.4)	87 (27.0)	53 (10.5)	18 (15.7)	0	28 (18.9)	0	320 (16.4)
Any critical site (inside patient zone) (% of all HSE)	57 (11.5)	30 (6.4)	29 (9.2)	12 (2.4)	21 (4.4)	31 (4.6)	213 (38.5)	1 (0.6)	100 (19.0)	0	494 (11.7)
Sterile equipment (% of HSE at critical site)	1 (1.8)	0	0	0	0	0	123 (57.8)	1 (100)	82 (82.0)	0	207 (41.9)
Invasive device access (% of HSE at critical site)	55 (96.5)	30 (100)	29 (100)	9 (75.0)	21 (100)	21 (67.7)	65 (30.5)	0	8 (8.0)	0	238 (48.2)
Mucous membrane (% of HSE at critical site)	1 (1.8)	0	0	3 (25.0)	0	0	0	0	0	0	4 (0.8)
Wound (% of HSE at critical site)	0	0	0	0	0	10 (32.3)	25 (11.7)	0	10 (10.0)	0	45 (9.1)
Infectious risk events	41	42	43	26	44	80	117	31	72	2	508
Patient colonization events	13	26	23	16	25	54	65	30	37	2	291
Patient infection events	38	16	20	10	19	26	52	1	35	0	217
Hand hygiene actions; n	8	13	14	7	11	9	15	4	11	5	97
Hand hygiene action at colonization event; n (% of patient colonization events, i.e. ‘adherence’)	0	3 (11.5)	2 (8.7)	1 (6.2)	3 (12.0)	2 (3.7)	0	2 (6.7)	1 (2.7)	0	14 (4.8)
Hand hygiene action at infection event; n (% of patient infection events, i.e. ‘adherence’)	0	0	0	0	0	0	2 (3.9)	0	1 (2.9)	NA	3 (1.4)
Average density of hand hygiene actions; n/hour	13.8	22.4	24.1	11.6	21.1	16.5	26.9	14.4	20.4	18.1	19.6
Mean duration of hand hygiene actions (SD); sec	8.6 (4.7)	14.9 (6.6)	22.2 (11.0)	18.8 (7.4)	11.7 (4.5)	9.2 (4.8)	10.5 (9.6)	16.3 (12.0)	7.9 (3.7)	11.6 (6.2)	13.2 (8.6)

Legend: *HSE* hand-to-surface exposure; *ICU* intensive care unit; *F* female; *M* male; *N* nurse; *P* physician; *NA* not applicable; *SD* standard deviation. Definition for *patient colonization event* and *patient infection event* s. main text

The mean and median duration of the 97 observed hand hygiene actions were 12.9 (SD, 8.7) and 11 (range, 2–48) seconds, respectively. Patient *colonization events* occurred overall 291 times, 139 for the left and 152 for the right hand. Patient *infection events* were observed overall 217 times, 103 for the left and 114 for the right hand. Importantly, 117 (61%) of *colonization events* and seven (2.3%) *infection events* occurred after HCWs touching their own body. HCWs touched themselves 439 times (10% of all HSE), including their clothes 165 (38%), personal protective equipment 21 (5%), their face 24 (6%), and remaining bare skin or hair 229 (52%) times; 13 (3%) times with gloved hands.

Hand hygiene occurred prior to 14 of the 191 *colonization events* and three of the 217 *infection events*, resulting in a hand hygiene ‘adherence’ of 5% and 1%, respectively.

## Discussion

This unique video observation and coding approach, that considers each single HSE by both HCW hands, revealed a surprising reality of transmission opportunities during real-world intensive care. The overall density of 14.2 HSE per minute with which HCWs’ hands touched surfaces during active patient care is high, suggesting that hands acquire and deposit – and thus likely transmit – potentially harmful microorganisms every 4 s onto patients and surfaces in the care environment. We identified sequences of particular interest for infection prevention, such as patient zone entries and transitions to critical sites, which each occurred roughly every 2 min of active patient care in an ICU. Hand hygiene was performed on average 19.6 times per hour, which equals one hand hygiene action every 3 min. It is not surprising that participants only sustained hand rubbing for a median of 11 s against the recommended 20–30 s [17]. In fact, if meeting the recommended duration for hand rubbing, almost one fifth of active patient care time would have been spent on this activity. Recent data indicating that 15 s might suffice are comforting in this respect [18].

The approach used in this study is in line with a human factors task analysis, whose underlying principle is to break down a task to study its individual elements [19]. In doing so, we aim to understand the factors that influence the way work is being done and, ultimately, what can be done to improve it [20, 21]. In doing so, the moments we report here are more frequent than those usually reported in direct hand hygiene observation studies. For example, tasks such as a dressing change are typically seen as constituting one single hand hygiene opportunity with an indication ‘Before clean/aseptic procedure’ before the task and ‘After body fluid exposure risk’ at the end of the task [10]. In the current approach, each care task is split into multiple HSEs, taking into account both mobile objects [22] and the HCWs own body, each scrutinized for potential hand contamination and transmission. Furthermore, traditional hand hygiene models are based on the assumption that surfaces within the patient zone are colonized primarily with the patient’s own flora. Our results [11], however, demonstrate that frequent transitions of hands into the patient zone without hand hygiene may lead to contamination of the patient zone with foreign microorganisms. Such lapses lead to an unsafe system state, which creates ambiguity [23] and may result in unintentional patient harm.

Our approach revealed further noteworthy realities. We considered the HCW’s own body as an ‘Outside patient zone’ surface. More than half of all HSE sequences (61%) from the “outside” to the “inside” patient zone were due to ‘self-contact’. Current hand hygiene guidelines often fail to address HCW self-contact as an indication for hand hygiene [17]. Hence, such HSE are usually ignored by observers. Second, much variation exists in whether HCWs perceive their professional apparel as a potential source of bacteria, leading to variations in hand hygiene [24]. Additionally, as described by Sax & Clack, relying on automatic, unconscious behaviors fuelled by “mental models” for routine tasks is inherent to the nature of human beings, allowing mental resources to be spared for more complex tasks [11]. This suggests that people often are unaware of what exactly their hands do while they are focused on the main task goal [11]. The average of 1.48 exposures per minute to a HCW’s own body is consistent with previous findings [25, 26]. However, with only 4.87 exposures per hour to “HCW Face”, our results differed from studies who found that face contact occurred up to 15–23 times per hour among students during 2-h lectures [26] or during office-type work [25]. Finally, glove use was frequent, representing one fifth of all HSE. Gloves represent mobile surfaces that transport microorganisms like bare hands. Further research could explore the nature of HSE during glove use to inform best practice for glove indications.

The “first-person view” of a head-mounted action camera provides the advantage of an unobstructed view of both hands and the surfaces they touch following the healthcare worker [27] even when leaving the immediate care area, neither of which can be guaranteed with a fixed-position camera. From anecdotal reports by the participants, their awareness of wearing a camera and their activity being registered waned quickly, suggesting a minor Hawthorne effect, yet this remains to be studied systematically. Contrary to concerns about video recording in acute care settings, we found that once healthcare workers, patients, and their relatives were informed of the study goals, objections to filming were rare. Video observation of hand hygiene behavior has been used before [28–31] but never from a first-person view and never to record HSEs.

Our approach has limitations. The analysis is limited to a small sample of healthcare workers in three ICUs and in consequence not representative for care in general. We do not expect, however, the main findings of frequent HSE to be categorically different. Furthermore, while the sequential analysis we report here considers only pairs of two consecutive HSE leading up to “colonization” or “infection” events, it is important to recognize that HSE occur in long sequential chains. The exact benefit of hand hygiene at any of these moments has not been considered in our current calculation, nor in the WHO ‘Five moments’ concept. In this line of thought, our approach might serve as basis for more advanced future transmission risk modelling. Our definition of a *colonization event* deviated from ‘Moment 1’ of the WHO hand hygiene concept by including any object within the patient zone, not only the patient. We did this intentionally to identify the transmission trajectories most likely leading to contamination of high-touch surfaces near the patient and ultimately, the patient. On a technical note, the specific software is expensive and its use requires expertise. Video coding is more time-consuming than live observation. Hence, before introducing this instrument into day-to-day practice beyond research, simplification and automation is a desirable next development step. Finally, the videos were coded by a single coder (MS) and supervised by a second person (LC) due to feasibility. The possibility to pause and rewind the video likely minimized the risk of miscoding.

In conclusion, our approach produced a valid video and coding instrument for analysis of detailed HSE trajectories. Using a head-mounted action camera and a comprehensive coding system, we could show for the first time in a fast-paced, real clinical setting how frequently healthcare workers’ hands touch surfaces, corroborating the fast spread of microorganisms in healthcare settings. Further development and use of this method may contribute to the design of more efficient preventive strategies.

## Conclusions

Using a new head-mounted action camera and a systematic coding tool, we could show for the first time how healthcare workers’ hands touch surfaces in a real-world clinical setting. This human factors approach to task analysis demonstrated the hand trajectories via which microorganisms can spread in healthcare and revealed that hand hygiene adherence is lower than usually reported by traditional on-site observations. This new instrument may assist in designing more efficient preventive strategies on an individual and systems level.
